# Sleep and Cognition in Depression– A Feasibility Study

**DOI:** 10.1192/bjo.2026.11221

**Published:** 2026-06-30

**Authors:** Jennifer Burgess, Alice Mitchell, Kirstie Anderson, Peter Gallagher, Hamish, McAllister-Williams

**Affiliations:** 1 Newcastle University, NewcastleuponTyne, United Kingdom; 2 Newcastle Hospitals NHS Foundation Trust, NewcastleuponTyne, United Kingdom; 3 Cumbria, Northumberland, Tyne and Wear NHS Foundation Trust, NewcastleuponTyne, United Kingdom

## Abstract

**Aims:**

Over 90% of patients with depression report troublesome sleep-wake disturbances, and patients with depression are more likely to have comorbid sleep disorders such as insomnia. They also display small but significant deficits in a variety of cognitive domains, and both sleep and cognitive problems can persist into remission. Sleep disturbances also adversely affect cognition, however due to heterogeneity of methods and results in the extant literature, further research is required to assess how sleep disturbances affect cognition in depression.

**Methods:**

Forty participants aged 18–65 with unipolar depression and 40 gender- and age-matched controls were recruited to this naturalistic observational study. Baseline measures of mood and sleep were completed, and participants then wore a triaxial accelerometer (actigraph) and completed a sleep diary. At the second study visit 14 days later participants completed a battery of neuropsychological tasks, including the Psychomotor Vigilance (PVT), Continuous Performance (CPT), Digit Symbol Substitution (DSST), and Trail-Making-Test (TMT).

**Results:**

Following exclusions, 34 participants in each group were included in the final analysis. In the depression group the average age was 49.5, 53% were female, and 94% White British Ethnicity. 44.1% were diagnosed with comorbid disorders (e.g. anxiety) and were prescribed a median 2 psychotropic medications. Eight patients were not currently depressed as assessed by the Hamilton Depression Rating Scale. The depression group were significantly more likely to report insomnia symptoms than controls, and although there was no difference in the average hours of night-time sleep (depression=6.39, control=6.69), the depression group had a significantly lower Sleep Regularity Index and more nights of short sleep (depression=6, control=4). There were no significant group differences between the primary outcomes PVT and CPT, however the depression group demonstrated significantly slower DSST and TMT-A performance. There were few significant correlations between sleep and cognitive variables, with the strongest observed between insomnia symptoms and slower DSST performance. Multiple regression analyses showed that insomnia symptoms, but not actigraph-measured hours of sleep, moderated the relationship between depression symptoms and PVT performance.

**Conclusion:**

In this study, participants with depression reported significantly more insomnia, and had a less regular sleep schedule than controls. There were no group differences on the primary outcome sustained attention tasks, but the depression group demonstrated significantly slower psychomotor processing. The relationship between depression symptoms and sustained attention was moderated by insomnia symptoms but not average hours of sleep.

